# FRET Imaging of Hemoglobin Concentration in *Plasmodium falciparum*-Infected Red Cells

**DOI:** 10.1371/journal.pone.0003780

**Published:** 2008-11-21

**Authors:** Alessandro Esposito, Teresa Tiffert, Jakob M. A. Mauritz, Simon Schlachter, Lawrence H. Bannister, Clemens F. Kaminski, Virgilio L. Lew

**Affiliations:** 1 Department of Chemical Engineering and Biotechnology, University of Cambridge, Cambridge, United Kingdom; 2 Department of Physiology, Development and Neuroscience, University of Cambridge, Cambridge, United Kingdom; 3 Centre for Ultrastructural Imaging, Kings College London, London, United Kingdom; 4 School of Advanced Optical Technologies, Max-Planck-Research Group, Division III, University of Erlangen-Nuremberg, Erlangen, Germany; Geroge Mason University, United States of America

## Abstract

**Background:**

During its intraerythrocytic asexual reproduction cycle *Plasmodium falciparum* consumes up to 80% of the host cell hemoglobin, in large excess over its metabolic needs. A model of the homeostasis of falciparum-infected red blood cells suggested an explanation based on the need to reduce the colloid-osmotic pressure within the host cell to prevent its premature lysis. Critical for this hypothesis was that the hemoglobin concentration within the host cell be progressively reduced from the trophozoite stage onwards.

**Methodology/Principal Findings:**

The experiments reported here were designed to test this hypothesis by direct measurements of the hemoglobin concentration in live, infected red cells. We developed a novel, non-invasive method to quantify the hemoglobin concentration in single cells, based on Förster resonance energy transfer between hemoglobin molecules and the fluorophore calcein. Fluorescence lifetime imaging allowed the quantitative mapping of the hemoglobin concentration within the cells. The average fluorescence lifetimes of uninfected cohorts was 270±30 ps (mean±SD; N = 45). In the cytoplasm of infected cells the fluorescence lifetime of calcein ranged from 290±20 ps for cells with ring stage parasites to 590±13 ps and 1050±60 ps for cells with young trophozoites and late stage trophozoite/ early schizonts, respectively. This was equivalent to reductions in hemoglobin concentration spanning the range from 7.3 to 2.3 mM, in line with the model predictions. An unexpected ancillary finding was the existence of a microdomain under the host cell membrane with reduced calcein quenching by hemoglobin in cells with mature trophozoite stage parasites.

**Conclusions/Significance:**

The results support the predictions of the colloid-osmotic hypothesis and provide a better understanding of the homeostasis of malaria-infected red cells. In addition, they revealed the existence of a distinct peripheral microdomain in the host cell with limited access to hemoglobin molecules indicating the concentration of substantial amounts of parasite-exported material.

## Introduction

*Plasmodium falciparum* (*Pf*) causes the most severe form of malaria. Over one third of the world population is estimated to be at risk [1]. During its intra-erythrocytic asexual reproduction cycle of about 48 hours, the parasite causes major changes in the metabolism and transport of the invaded red blood cell (RBC) [2], [3]. A previous analysis of the homeostasis of the infected red blood cells (IRBCs) using a mathematical model developed *ad hoc*, combined with experimental results that showed that the osmotic fragility of IRBCs becomes progressively increased throughout the asexual cycle, suggested that IRBCs are prone to premature haemolysis, and that the parasite may prevent lysis by reducing the colloid osmotic pressure within the host cell [4]. It is well known that *Plasmodium falciparum* consumes up to 70–80% of the host cell hemoglobin (Hb) [5], but uses only up to 16% of the produced amino acids for *de novo* protein synthesis [6]. The bulk of the amino acids are released to the extracellular medium via “new permeation pathways” (NPPs) present in the host cell membrane and induced by the parasite [2], [3]. According to the “colloid-osmotic” hypothesis [4], [7], the reason why the parasite ingests and digests Hb in such excess is to reduce the colloid-osmotic pressure within the host and thus prevent premature lysis. For this hypothesis to be tenable it is critical that not only the Hb content of the IRBC is reduced, but also its concentration ([Hb]), because colloid-osmosis is determined by the concentration gradient of the impermeant solutes across the water-permeable red cell membrane. Previous indirect estimates of [Hb] have given contradictory results. In electronmicrographs of IRBCs, the electron density of the material in the cytoplasm of the host RBC, assumed to reflect Hb concentration, was found by different authors either identical to [8], or lower than that of neighbouring uninfected RBCs [9], without any clear methodological explanation for that difference. Measurements in live, unprocessed specimens are clearly preferable for estimates of concentration likely to be affected by sample processing procedures that may alter cell volumes differently in cells with normal and altered permeabilities.

We report here direct measurements of Hb concentration within the cytosol of live IRBCs using a novel technique based on fluorescence lifetime imaging (FLIM), to test the validity of the colloid-osmotic hypothesis. Because of the spectral characteristics of the heme chromophore [8], hemoglobin is a powerful quencher of any fluorophore with fluorescence emission spectra peaking below 600 nm. We envisaged that the primary cause of fluorescence quenching could be Förster resonance energy transfer (FRET) from the donor fluorophore (calcein, in this work) to the acceptor heme chromophore induced by hemoglobin molecular crowding. FLIM combines the high spatial resolution of the confocal detection scheme, necessary to discriminate between host- and parasite- compartments, with the high fluorescence lifetime resolution provided by time-correlated single photon counting (TCSPC) [9]. Such a combination is needed to cover the relevant range of hemoglobin concentration (2–7 mM) with sufficient spatial resolution. Mapping absorption of Hb within the infected RBC may appear a feasible alternative for [Hb] quantification. However, the need for simultaneous estimation of cell thickness at each pixel together with the requirement to discriminate unambiguously between RBC and *Pf* compartments with sufficient resolution makes this approach less practicable at present. FRET imaged by FLIM is one of the most robust techniques to detect molecular interactions with comparatively high spatial resolution [10], [11] making this the method of choice to quantify [Hb] in live cells.

## Results

### Model predictions

**Figure 1:**
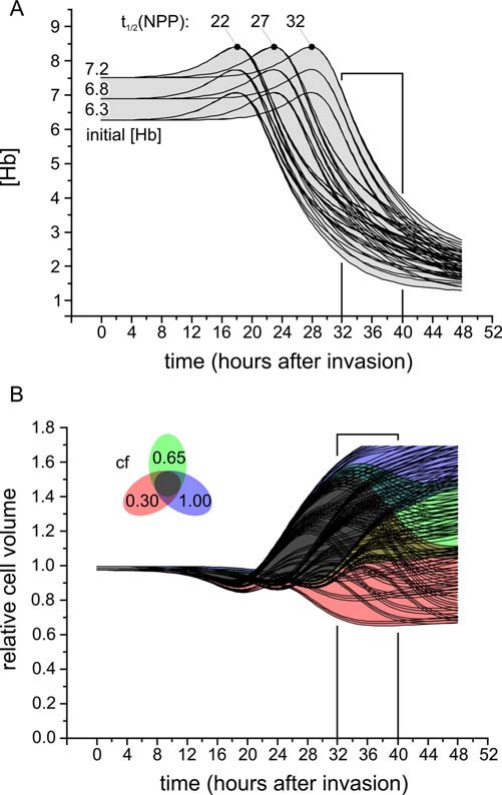
Predicted changes in [Hb] (*A*) and relative cell volume (B) as a function of time post-invasion. The different curves reflect variations in parameter values within ±1SD of experimentally reported values: t_1/2_(NPP) and t_1/2_(Hb) were varied over the range of 27±5 hours [12] and 32±6 hours [6], respectively; the initial hemoglobin concentration and the maximal fraction of digested hemoglobin were set to 6.8±0.5 mM and 80±10% [5], respectively. The coupling factor (*cf*) between the volume of cytosol ingested by the parasite and its volume-growth was set at 0.30, 0.65 and 1.00. The vertical column singles out the 32 to 40 hour post-invasion period of the cell samples analysed here. Volume is reported relative to initial red cell volume.

Figure 1 shows the predicted changes in [Hb] (Fig. 1A) and in relative cell volume (Fig. 1B) of IRBCs as a function of time post-invasion. The different curves explore parameter values within a standard deviation (SD) of the mean values reported in the literature [6], [12]. The four parameters varied were: average time of appearance of the NPPs or t_1/2_(NPP), average time for Hb digestion or t_1/2_(Hb), initial hemoglobin concentration and maximal fraction of digested hemoglobin. The envelopes of the curves, grey in Fig. 1 A and coloured in Fig. 1 B, represent the 99% percentiles of the joint distribution for the four parameters. Additionally, Fig. 1 B shows the effect of variations in parasite volume growth, modelled by adjusting the value of a coupling factor (*cf*) that links the volume of ingested cytosol to that of the parasite [7].

According to model predictions, the vertical bar singles out a period of mature parasite growth with a high probability of detecting substantial reductions in Hb concentration within the host cell cytoplasm (Fig. 1A), from normal values of about 7 mM in uninfected or ring-stage infected red cells down to ∼4 mM in cells with mid-age trophozoites and 2–3 mM in schizont-containing cells, regardless of the IRBC volume (Fig. 1B).

### Quenching of calcein via FRET

**Figure 2:**
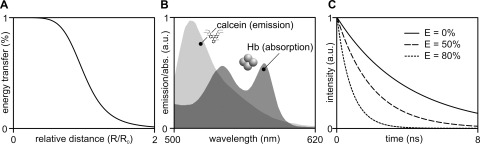
Fluorescence lifetime and FRET. A) Curve illustrating the strong dependence of energy transfer efficiency on the relative distance between donor and acceptor fluorophores. B) Spectral overlap between the emission spectrum of a donor and the absorption spectrum of an acceptor fluorophore such as calcein and heme on which FRET depends (*abs.*, absorption; *a.u.*, arbitrary units). C) Predicted fluorescence decay of a fluorophore such as calcein, in the presence of 0% (*solid line*), 50% and 80% (*dashed lines*) FRET efficiency.

FRET is the non-radiative transfer of energy from a donor fluorophore to an acceptor chromophore [13] (see Supplementary Text S1). The efficiency of energy transfer depends on the inverse of the sixth power of the intermolecular distance. At the so called Förster distance (R_0_, see Fig. 2A), the energy transferred from donor to acceptor is 50%. The Förster distance depends on the spectral properties of donor and acceptor (Fig. 2B) and it was estimated to be ∼4.1 nm for the calcein-heme pair. Interestingly, at physiological heme concentrations (∼28 mM; 1 Hb molecule = 4 heme moieties) there is always a significant probability to have acceptors in close proximity (∼R_0_) of a donor. The critical hemoglobin concentration ([Hb]_0_), i.e., the concentration at which 76% of energy is transferred [13], can be thus estimated to be about 1.7 mM (see Supplementary Text S1). FRET decreases the fluorescence lifetime of a donor fluorophore proportionally to the FRET efficiency (Fig. 2 C), and therefore it may be expected that FRET induced by molecular crowding [14] can be used to map [Hb] in living cells. This hypothesis was initially tested *in vitro*.

**Figure 3:**
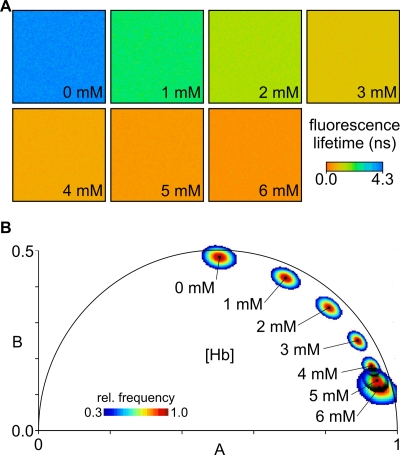
Quenching of calcein by hemoglobin. A) Images show the decrease in fluorescence lifetime of calcein (100 µM) with hemoglobin concentration (0 to 6 mM) in RBC lysates. Fluorescence lifetimes are easily distinguishable in the 0–4 mM range of [Hb]. B) Same calibration data are represented in the phasor space. The centroids of phasor distributions are also shown in figure 8 (circles). Rel. frequency: relative frequency.

**Figure 4:**
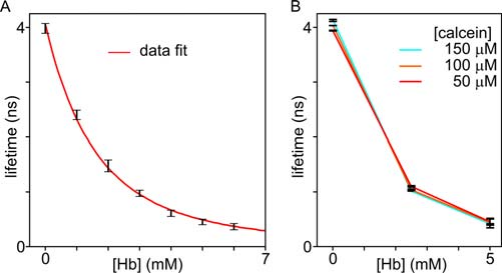
Quenching of calcein at different calcein and hemoglobin concentrations. A) Average fluorescence lifetime *versus* [Hb] (mean of three experiments). Experimental values were fitted by equation S6. B) Hb-dependent quenching of calcein at different calcein concentrations (50, 100 and 150 µM).

Fig. 3 shows fluorescence images of RBC lysates in the presence of calcein (100 µM) and their representation as phasor plots [15]. Lysate dilutions rendered Hb concentrations between 0 and 6 mM. The average fluorescence lifetimes are shown in Fig. 4A: the fluorescence lifetime of calcein in the absence of hemoglobin was 4080±10 ps and the [Hb]-dependent quenching of calcein appeared to be consistent with the proposed model (eq. S2).

Calcein is known to exhibit self-quenching at high (millimolar) concentrations [16], and it has also been suggested that self-quenching can occur at concentrations as low as 3 µM [17]. Self-quenching may result in either a decrease or increase in fluorescence lifetime, and could become a serious impediment for [Hb] measurements. It was therefore important to test for possible self-quenching effects within the range of calcein concentrations intended for intact cell measurements (<150 µM). *In vitro* tests showed that the maximal variation in calcein fluorescence lifetime within the 10–500 µM concentration range was ∼70 ps (data not shown) in phosphate buffer saline, growth medium and solution *A*. Fig. 4B shows that no significant de-quenching of calcein occurred at decreasing calcein concentrations (150 µM, 100 µM and 50 µM). Therefore, calcein can be used for the quantitative detection of hemoglobin concentration within the cytosol domain of infected red cells.

**Figure 5:**
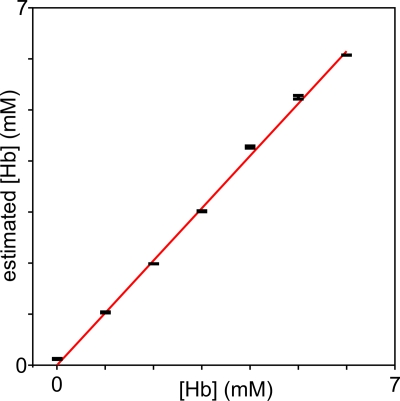
Measurement of [Hb]_0_. [Hb], estimated from eq. 1, is plotted as a function of measured [Hb] (intercept set to zero). The parameter [Hb]_0_ was estimated from the slope of the linear fit, as explained in the text. Bars are standard deviation from measurements performed in triplicates.

The calibration data shown in Fig. 3 was then fitted with eq. 1 to render estimated [Hb] values. Figure 5 shows the relation between estimated and measured [Hb]. The slope rendered [Hb]_0_ equal to 1.70±0.02 mM, a value in good agreement with the predicted one.

The results so far confirm that the prime mechanism for fluorescence quenching of calcein loaded into RBCs is dynamic quenching mediated by FRET via the non-fluorescent heme chromophore. The use of FRET induced by hemoglobin molecular crowding may thus be applied to report [Hb]. The lack of specificity of this mechanism may explain the quenching observed with other fluorophores emitting below 600 nm such as Fura-2, Indo-1 and Fluo-4 [18], [19].

### Lifetime imaging of normal, uninfected red blood cells

**Figure 6:**
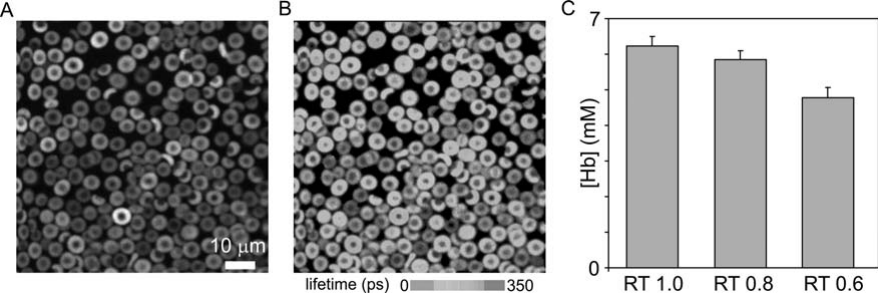
Fluorescence lifetime imaging of calcein loaded red blood cells. *A*) Fluorescence emission (520–570 nm). *B*) Fluorescence lifetime of calcein showing a decrease from about 4 ns at zero [Hb] down to 250 ps in intact uninfected RBCs. Note the variation in fluorescence intensity and fluorescence lifetime among the cells. These reflect the normal distribution of incorporated calcein and [Hb] in red cells [19], [41]. C) [Hb] measured by fluorescence lifetime in RBCs whose [Hb] was reduced by equilibration in hypotonic media (RT: relative tonicity).

Fig. 6 shows a low magnification image of RBCs acquired by TCSPC. The high physiological concentration of hemoglobin renders the method only moderately sensitive to [Hb] variations near physiological conditions. Normal RBCs (Fig. 6A–B) exhibited an average fluorescence decay time of (mean±SD) 250±20 ps (N = 37) corresponding to a [Hb] of 6.3±0.3 mM. When the RBCs were swollen in solutions of lower relative tonicity (RT), fluorescence lifetime changes were clearly detected (Fig. 6C). At RTs of 0.8 and 0.6, the fluorescence lifetime of calcein increased to 280±20 ps (N = 38) and 380±40 ps (N = 30), respectively, equivalent to Hb concentrations of 5.9±0.3 mM and 4.8±0.3 mM. At lower RTs significant lysis interfered with the [Hb] estimates and could not be pursued.

### Lifetime imaging of parasitized RBCs

**Figure 7:**
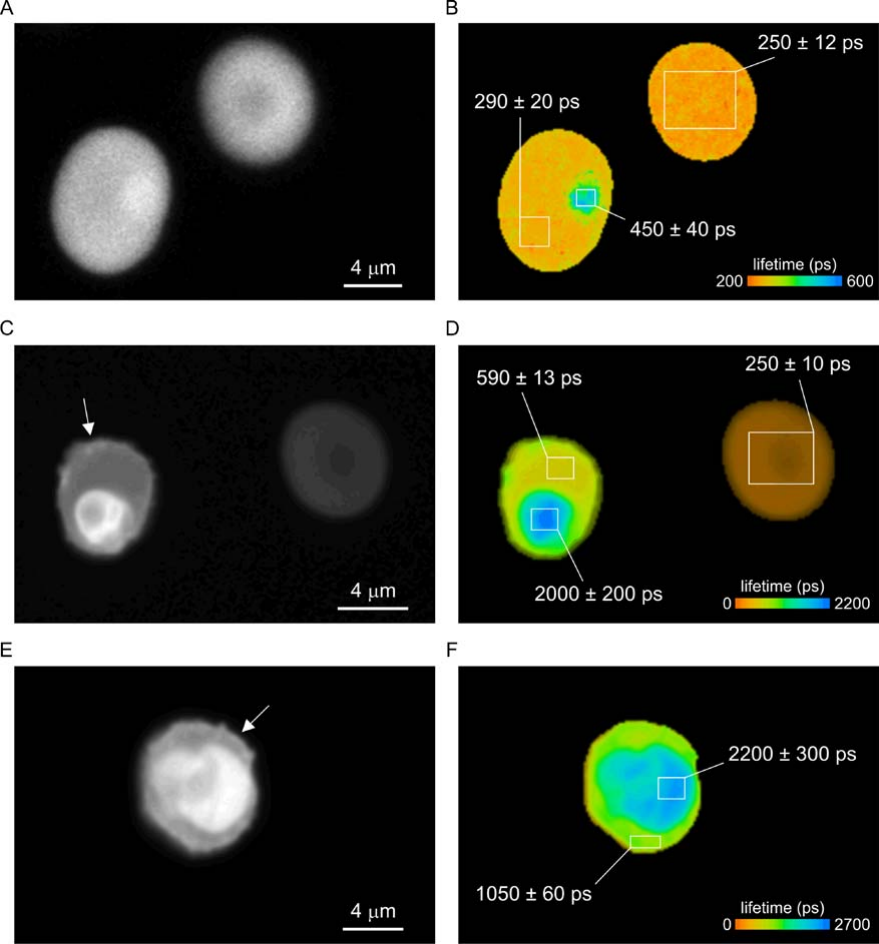
Lifetime images of infected red blood cells. Panels A, C and E show fluorescence intensity images of an uninfected RBC (*A and C*, *right*), a ring stage (*A*, *left*), a young trophozoite (*C*, *left*) and a mature trophozoite/early schizont (*E*). Arrows in panel C and E indicate the appearance of a bright edge in the IRBCs. The correspondent fluorescence lifetime images (*B*, *D and F*) reveal a significant increase in fluorescent lifetime in the host cytosol of infected cells relative to that in uninfected RBCs reflecting a decreased hemoglobin concentration.

Figure 7 shows representative images of infected and non-infected RBCs. Non-infected cells showed homogeneous fluorescence intensity and fluorescence lifetime. The fluorescence lifetime of calcein averaged over the uninfected cells in these RBCs was 250±12 ps (Fig. 7 B, right) and 250±10 ps (Fig. 7 D, right). In the cytoplasm of IRBCs the fluorescence lifetime of calcein ranged from 290±20 ps for a ring-IRBC (Fig. 7 B, left) to 590±13 ps (Fig. 7 D, left) and 1050±60 ps (Fig. 5 F) for a young and a late stage trophozoite/early schizont, respectively.

**Figure 8:**
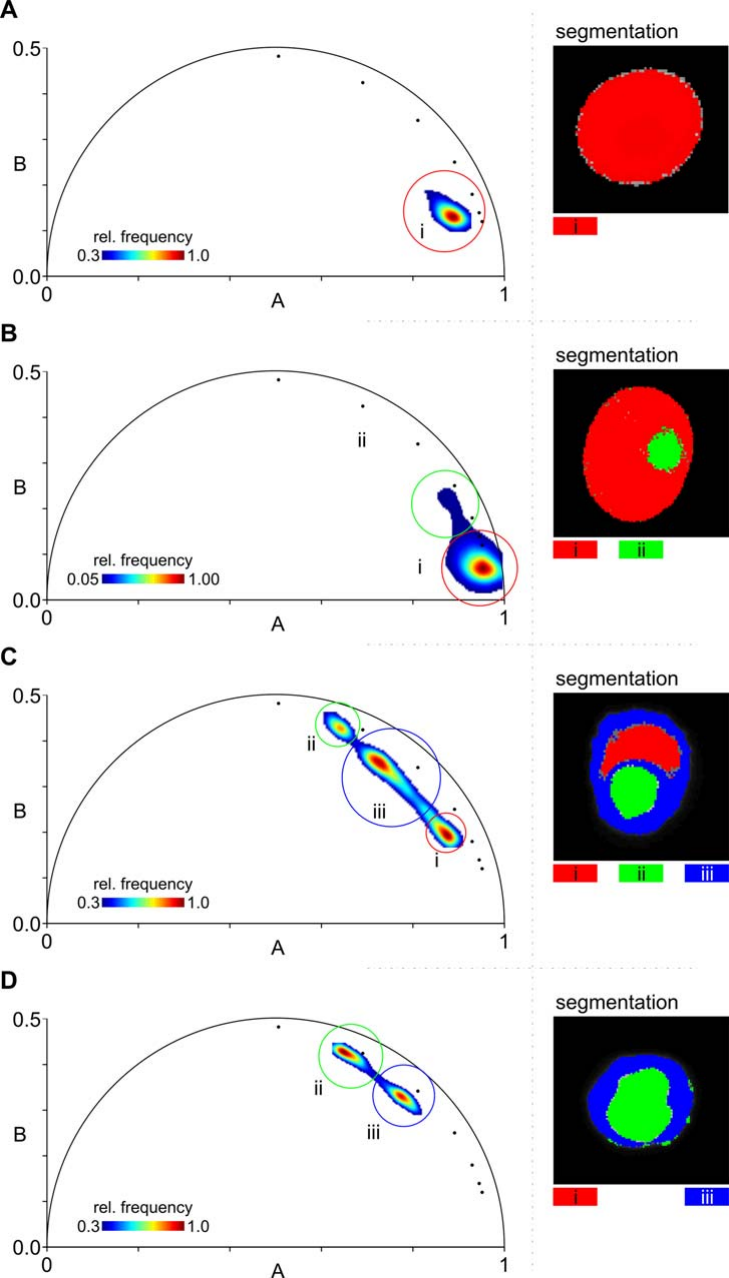
Segmentation of lifetime images by AB- (phasor) plots. AB-plots and corresponding segmented regions of the cells shown in Fig. 5. The segmented regions are shown on cells in green, red and blue colours on the right. A) Uninfected RBC (Fig. 5 B right). B) Ring-IRBC (Fig. 5 B left). C) Young trophozoite-IRBC (Fig. 5 D left). D) Mature trophozoite- / early schizont-IRBC (Fig. 5 E). The centroids of the phasor distributions from the calibration data (Fig. 3) are indicated by the small black circles ranging clockwise along the arc from 0 mM to 6 mM. Three segmented regions are shown in the AB-plots by coloured circles: i) RBC cytosol (red), ii) malaria parasite (green) and iii) a mostly peripheral compartment (blue) within trophozoites / schizonts: see text. Rel. frequency: relative frequency.

In order to discriminate between regions of the RBC where the parasite is located and the remaining cytoplasm, the lifetime images were mapped to the phasor space resulting in AB-plots (Figure 8) where pixels with similar lifetimes cluster together [15], [20], [21]. These clusters can be selected and the corresponding regions segmented. Uninfected RBCs always exhibited single clusters (Fig. 8A) with occasional tails that converged to the fluorescence background (data not shown). All infected cells exhibited two or three clusters with the one at lower lifetimes corresponding to the RBC cytoplasm. Fig. 8B shows an IRBC containing a ring-stage parasite; here, the parasite-related cluster (ii) is less defined (note the different scale) because of the small region occupied by the ring relative to the RBC cytoplasm (i). IRBCs containing early trophozoites (Fig. 8C) and late trophozoites / early schizonts (Fig. 8C–D) typically exhibit three clusters corresponding to (i) RBC cytoplasm, (ii) the parasite and (iii) two other regions within the IRBC, one immediately encircling the parasite and the other forming a peripheral zone beneath the IRBC plasma membrane. Late trophozoites / early schizonts (Fig. 8D) often exhibit only two regions with comparatively low [Hb] estimates. The cytosolic cluster with the highest [Hb] in each cell was used to estimate cytosolic [Hb].

### Hemoglobin concentration in parasitized cells

**Figure 9:**
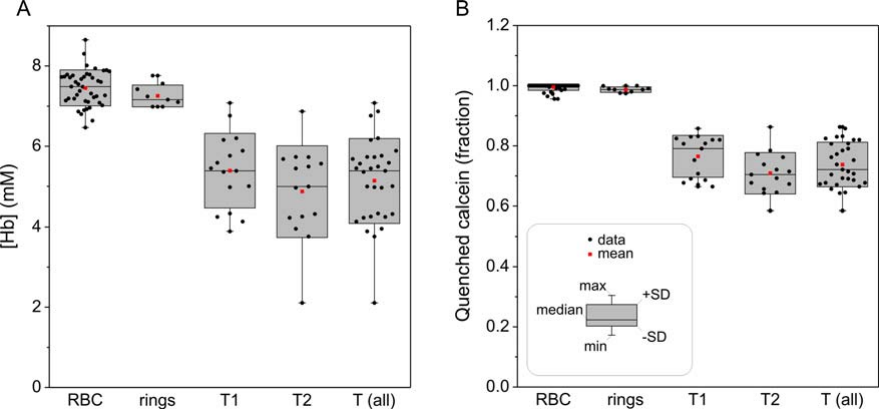
Statistical analysis of cytosolic [Hb] (A) and quenched calcein fractions (B). Columns report uninfected cohorts (*RBC*), IRBCs containing ring-stage parasites (*ring*), trophozoite stage parasites (*T1*) and trophozoite-schizont stage parasites (*T2*), and pooled T1+T2 data (*T all*). The values shown in the statistical box charts were measured in the host cytoplasm from the phasor-assisted segmentation.

The average fluorescence lifetimes of uninfected cohorts was 270±30 ps (N = 43), and of IRBCs with ring-stage parasites was 267±13 ps (N = 10). There was no statistical difference between these two groups. The distribution of fluorescence lifetimes and corresponding [Hb] was much wider in trophozoite/schizont-containing IRBCs (550±230 ps, N = 30). Figure 9 shows the cumulative statistics for the estimates of cytosolic [Hb]. Uninfected RBCs and ring-containing IRBCs exhibited statistically similar hemoglobin concentrations of 7.5±0.4 mM (N = 43) and 7.3±0.3 mM (N = 10), respectively. In both cases, the fraction of quenched calcein was close to 1 (RBCs: 1.00±0.01; rings: 0.99±0.01), i.e., all calcein molecules are undergoing energy transfer. RBCs containing post-ring stage parasites were classified into two classes: young trophozoites (T1) and mature trophozoites/early schizonts (T2). Without specific stage and viability markers, it is impossible to attribute a more precise developmental stage and viability condition to individually selected IRBCs under confocal observation. The visual inspection of the sample allows the classification of the IRBCs accordingly to their morphology or the presence of hemozoin aggregates. The trophozoite/schizont attribution based on fluorescent size outlines (Fig. 7) and degree of pigment aggregation can only be considered a rough approximation. Notwithstanding this uncertainty, the results in Fig. 9A unambiguously demonstrate the reduction in the Hb concentration within the cytosol of IRBCs with mature-stage parasites. In fractions T1 and T2, the hemoglobin concentrations in host-cell cytoplasm were 5.4±0.9 mM (N = 16) and 4.9±1.1 mM (N = 15), respectively, with a pooled average of 5.1±1.1 mM (N = 31; T (all)). The scatter of [Hb] values was much wider in trophozoite-infected than in uninfected red cells, ranging between 2.1 and 7.1 mM (Fig. 9A, T (all)). The fraction of quenched calcein (Fig. 9B) was 0.77±0.07 (T1), 0.71±0.07 (T2) and 0.74±0.07 (T (all)) suggesting a moderate compartmentalization of calcein; about 25% of calcein molecules in the cytosol of torophozoite-infected RBCs was not found in close proximity of heme moieties in spite of the high [Hb] still detected. Two thirds of trophozoite-infected RBCs (T1) showed a third cluster, but only 20% of the cells in the fraction T2 showed three distinct clusters in the phasor space. Analysis of values from the third cluster indicated slightly lower values of [Hb] (4.3±1.1, N = 13) spanning a range between 1.7 and 5.8 mM and a significantly higher compartmentalization (0.61±0.09). The parasite compartment showed an apparent [Hb] value of 3±1 mM and a fraction of quenched calcein of 0.46±0.08. These values of course relate to sampling which included IRBC cytosol below and above the parasite as well as the parasite itself.

## Discussion

The experiments presented here were designed to test a critical prediction of the colloid-osmotic hypothesis (Fig. 1A), that parasite development is accompanied by a progressive decline in hemoglobin concentration in the host cell cytoplasm [4], [7]. We discuss first some aspects of the methodological approach applied in this investigation. As highlighted in the Introduction, the optimal way to test this hypothesis is to map the local hemoglobin concentration in the cytoplasm of intact, live, infected red blood cells. The method developed and applied for such measurements here was based on fluorescence quenching of the cell-incorporated fluorophore calcein. Both *in vitro* (lysate) and *in situ* (intact RBC) calibrations using the fluorophore calcein (Figs. 3 and 4) proved that fluorescence lifetime is sensitive to the local hemoglobin concentration. The observed reductions in fluorescence lifetimes proved that molecular crowding induced FRET is indeed the main cause of quenching and that it can be applied to estimate local [Hb].

Confocal detection together with TCSPC provided the spatial resolution required for the quantitative mapping of [Hb] in the host cell cytosol. However, the limited number of photons per pixel (∼300) that could be collected in 60 s of acquisition time made image binning (5×5 pixels, see Fig. 7) necessary to reach the high signal-to-noise ratios required to fit multi-parameter fitting curves (eq. S2 and eq. S6 [22]). This standard image processing blurs structures in the sample and reduces the contrast in the cytosol of infected RBCs. The analysis of the average fluorescence lifetimes (eq. S6) indicated a significant reduction in [Hb] (data not shown), but did not provide the necessary spatial resolution. We therefore used a recent technique representing lifetime data based on AB-plots (or phasors) which map the time-resolved fluorescence decays to a linear bidimensional space where trajectories of clusters can be interpreted [15], [20]. AB-plots are particularly useful for fluorescence lifetime data segmentation [21] because regions of the sample with similar fluorescence lifetimes will cluster together in the phasor space.

The results showed that the [Hb] in the host cytosol of parasitized cells decreased from values around 7.5 mM in the RBCs used for cultures, to values between 2.1–7.1 mM in IRBCs, well within the range predicted by the numerical model (Fig. 1A). This approach has allowed us to present a more detailed view of the molecular processes occurring in a living IRBC. In fact, one of the most important advantages of this representation is the possibility of segmenting regions of the sample that exhibit different fluorescence lifetime prior to any data fitting [21]. Thanks to this strategy, it was possible to discriminate clearly between parasite and RBC cytoplasm. The photons collected from the segmented regions (10^5^–10^8^) provided the signal-to-noise ratios required to fit the proposed physical model (eq. 1) and to demonstrate the reduction in cytosolic [Hb]. The present results, documenting progressive dilution of [Hb] as the parasite matures, highlight the importance of studying live, unprocessed cell samples when evaluating homeostatic parameters. After submission of our manuscript a paper by Park and colleagues [23] appeared documenting an overall decline in host haemoglobin concentration with parasite maturation similar to the one reported here, by applying a combination of tomographic phase microscopy and difraction phase microscopy. The main prediction of the colloidosmotic hypothesis is thus supported by two independent observations obtained with different methods, both on live cell samples.

A surprising finding in the phasor analysis was the presence of a component with distinctive properties in IRBCs with mature parasites. Edge effects caused by RBC motion artefacts can be excluded because healthy RBCs show, at worse, a very narrow edge defined by slightly different lifetimes, which can be attributed to the mixing of calcein lifetime with background photons, resulting in faint tails in the phasor phase (Fig. 8). The distinct appearance of the three clusters in phasor diagrams of trophozoite- IRBCs (see Fig. 8C) suggests the formation of microdomains within the host cell cytoplasmic environment, in which hemoglobin appears largely excluded from close contact with calcein. In young trophozoites these domains are seen mainly as an extensive peripheral zone beneath the IRBC plasma membrane, with a similar though narrower zone around the parasite. In more mature IRBCs where the parasite is larger, the two regions coalesce, with no intervening Hb-rich zone (Fig. 8 D). This change is paralleled by an increased compartmentalization measured for calcein.

**Figure 10:**
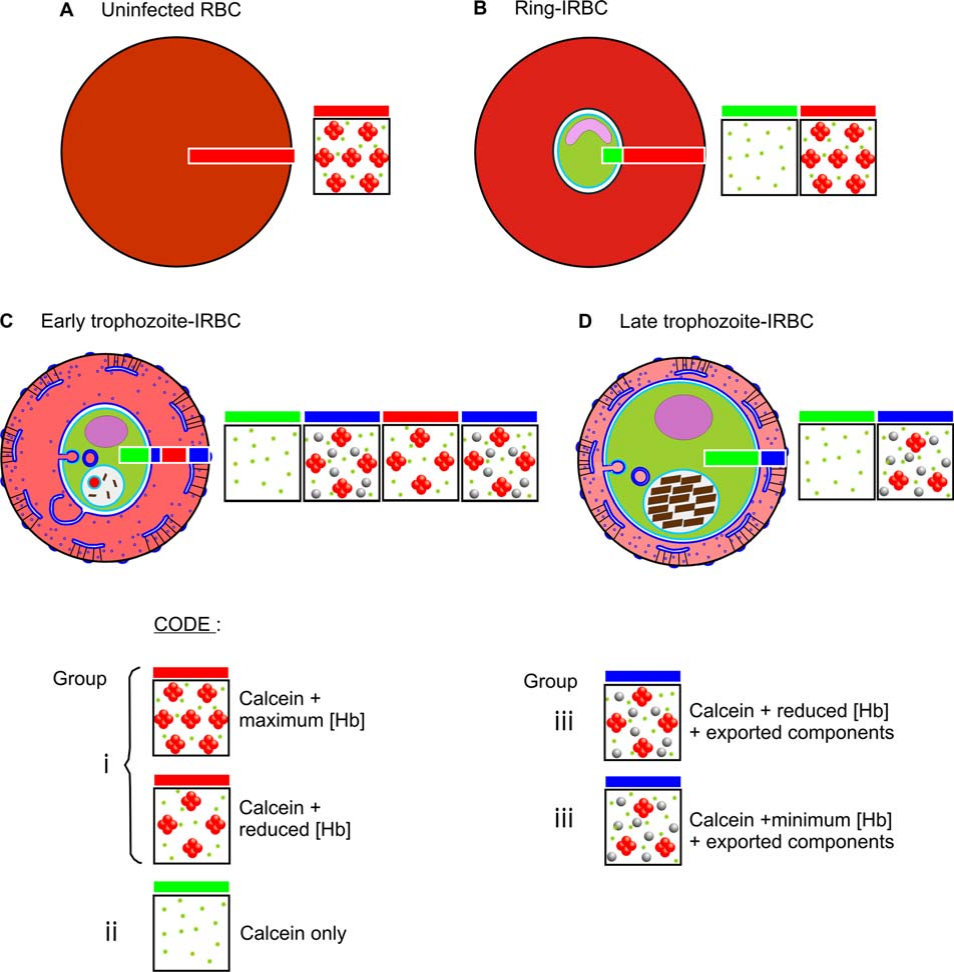
Interpretative diagram illustrating the observed [Hb] and compartmentation effects throughout parasite development. The boxes represent the state of the RBC / IRBC cytosol as determined by FRET, with horizontal red, blue and green bars, as for Figure 8.

These developments are likely to reflect localized changes induced in the IRBC by the parasite (see Fig. 10), including the export and assembly of molecules destined for Maurer's clefts, knobs and other parasite-derived structures in the peripheral zone of the host cell [24]–[27]. We note that also the fluorescence intensity of calcein appears higher at the cell periphery of trophozoite containing IRBCs (arrows in fig. 7C and E), but not in uninfected RBCs (Fig. 7 A and C, right) or ring-stage IRBCs (Fig. 7A, left), supporting the intepretration of significant calcein de-quenching beneath the membrane surface. Likewise, the traffic of parasite proteins from the parasitophorous vacuole surrounding the trophozoite is intense at this stage [28], and this may be related to the altered region close to the parasite surface. The model parameters from which the colloidosmotic hypothesis was derived ensure maintenance or rapid restoration of osmotic equilibria between host cell cytoplasm and extracellular medium. Could the Hb-restricted microdomain under the host cell membrane interfere with such equilibria? It is well established that isotonic sorbitol or alanine retain their lytic potential of IRBCs with parasites of all stages beyond the ring stage indicating that the Hb-restricted domain under the membrane represents no limiting permeability barrier to NPP-mediated or water fluxes. Therefore, any domains between the bulk Hb-containing cytosol of the host cell and the external medium cannot alter the way in which osmotic equilibria are maintained or restored between these two compartments.

In conclusion, the present results demonstrate that the well known reduction in Hb content of malaria-infected RBCs is accompanied by a concomitant reduction in Hb concentration, providing strong support for the colloid-osmotic interpretation of excess Hb consumption. They also reveal the existence of a submembrane compartment where Hb is partially excluded, locally diluted, or both. The molecular basis of this change awaits a more detailed analysis of the complex parasite-host cell interaction.

## Materials and Methods

### Chemicals

All chemicals were analytical reagent quality. Calcein-AM was from Invitrogen Ltd., Paisley, UK. Plasmagel was from Bellon, Neuilly Sur Seine, France. EGTA, Hepes, glucose, inosine, pyruvate, DMSO, RPMI 1640 medium, gentamicin sulphate, glutamine, free calcein, triton X-100, bovine serum albumin (BSA) and all common salts were from Sigma-Aldrich Company Ltd., Gillingham, UK. Human blood and serum used in cultures were from the National Blood Service (UK).

### Cultures

Two *P. falciparum* clones (ITO4 and A4BC6, kindly provided by B.C. Elford, Institute of Molecular Medicine, Oxford, UK) [29] were cultured separately in human erythrocytes under a low-oxygen atmosphere by standard methods [30]. The culture medium was RPMI 1640 supplemented with 40 mM HEPES, 25 mg/l gentamicin sulphate, 10 mM D-glucose, 2 mM glutamine and 8.5% (vol/vol) pooled human serum. Parasite development and replication were assessed in cultures by microscopic examination of Giemsa-stained thin blood smears, as reported before [31]. Parasitised RBCs were harvested from cultures immediately prior to experimentation.

### Solutions

Solution *A* contained (in mM): NaCl, 145; KCl, 3; Na-HEPES (pH 7.5 at 37°C), 10; MgCl_2_, 0.15. Solution *AE*: as *A* plus 0.1 mM Na-EGTA. Solution *AIP*: as *A* plus 5 mM inosine and 5 mM pyruvate. Calcein-AM was used as a stock solution of 0.7 mM in DMSO. The stock solution of free calcein was 10 mM in DMSO.

### Calcein loading

Cell samples from malaria cultures were washed once in *AIP*, resuspended at 2% haematocrit (Hct) in the same medium and incubated at 37°C for 20 minutes with calcein-AM at a concentration of about 120 µmol per litre RBCs, assuming 100% free calcein incorporation into the cells. Inosine in the medium was used as a glycolytic substrate and pyruvate to bypass the glycolytic block generated by the release of formaldehyde during the breakdown of the acetoxymethyl ester that remains fully retained by the cells [32], [33]. After incubation, the cells were washed twice by centrifugation and resuspension in *AIP*; ∼4·10^6^ cells were then transferred to a chamber with glass bottom coated with poly-L-lysine (#1.0, MatTek Corp., Ashland, MA, USA) and further incubated at 37°C for 60 minutes to allow adherence to the coverslip. The supernatant was then exchanged with fresh solution *AE* supplemented with 1% BSA and the chamber transferred to the stage microscope for imaging.

### Preparation of lysate for [Hb] calibrations

Venous blood (5–10 ml) from healthy volunteers was drawn, after written consent, into syringes containing EGTA. The cells were immediately spun (1500 g, 10 min) to separate the plasma, washed twice by centrifugation (1500 g, 5 min) and resuspension in 10 volumes of solution *AE* to remove Ca^2+^ loosely bound to the cells [34], and twice more in solution *A* to remove EGTA from the medium. Supernatant and buffy coat were removed after each centrifugation. After the washes, the cells were packed by centrifugation (11 000 g, 3 min) and lysed by addition of Triton X-100 (1% vol/vol). The original [Hb] in the lysate was estimated by optical absorption at 415 nm in diluted samples, giving a [Hb] value for the original undiluted lysate in the range of 6.5 to 7.0 mM. Suitable Hb concentrations for calibration were obtained by diluting the concentrated lysate with solution *A*. Free calcein was added to the Hb samples from the 10 mM stock in DMSO to render the indicated final calcein concentrations. Further tests of the reliability of [Hb] estimates in intact cells were carried out on calcein-loaded RBCs exposed to hypotonic media to vary their [Hb]. Hypotonic media were prepared in the pre-lytic 0.6 to 1 range of relative tonicities by dilution of solution *A* with double distilled water.

### Microscopy

Fluorescence lifetime imaging microscopy was performed with the use of an in-house developed confocal microscope based on a confocal microscope Olympus FluoView 300 (Olympus UK Ltd, Watford, UK) coupled with a supercontinuum laser source (SC450, Fianium, Southampton, UK) [35]. The SC450 provides ∼10 ps pulses at a repetition rate of 40 MHz, suitable for TCSPC. The system was upgraded with a PMC-100-20 photomultiplier tube and a SPC-830 board for time-correlated single-photon counting, both by Becker & Hickl GmbH (Berlin, Germany). Calcein was excited at 485 nm and fluorescence emission was collected over the 520–570 nm range. TCSPC data was analysed with SPCImage software (Becker & Hickl GmbH) with a three-exponential decay and scatter light fitting to provide the average lifetime shown in Figures 3A, 4, 6B and 7. Average count rates were kept below 10^5^ counts per second in order to avoid pulse pile-up. All images were acquired at room temperature with an oil immersion 60× objective and a large (300 µm) pinhole in order to collect enough photons during the 60 s acquisition time.

### Data analysis

The fluorescence images were segmented in the phasor space by performing a sine (A) and cosine (B) transformation of the TCSPC data [15], [20]. The AB-plots were generated by in-house developed software programmed in Matlab (The MathWorks Inc., Novi, MI, USA). Photons from each segmented region were binned together and the following equation was fitted by iterative reconvolution [36]:
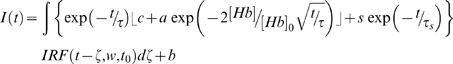
(1)


This represents the decay of calcein starting at *t_0_*, quenched by freely diffusing hemoglobin in the presence of unquenched donor (of lifetime τ) and scatter (τ_S_ = 10 ps), convolved with the instrument response function (IRF) and in the presence of background (*b*). *a*, *b*, *c* and *s* are absolute amplitudes of the different components. The best fit was found minimizing the cost function proposed by Awaya [37] in order to minimize biases generated by the presence of data bins with low counts. The molecular fraction of quenched calcein (*f_CA-Hb_*) can be inferred by the following equation:
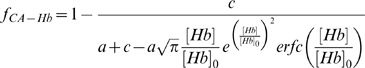
(2)


Additional information on FRET induced by molecular crowding and on the data analysis is provided in the Supplementary Text S1 and in the literature [13], [14], [38]–[40].

### Numerical computation

The Förster radius of calcein-heme chromophores was computed by numerical integration of the fluorescence emission spectrum of calcein and the corrected absorption spectrum of oxy-hemoglobin obtained by Invitrogen Ltd (http://probes.invitrogen.com) and the Oregon Medical Laser Center (http://omlc.ogi.edu), respectively; numerical integration were performed with Mathematica (Wolfram Research Europe Ltd., Long Hanborough, UK; see http://laser.cheng.cam.ac.uk). Model predictions for the homeostasis of IRBCs were performed with the computational code of the IRCM software [4], [7] integrated in an application developed in Matlab to simulate the heterogeneity of parameter values (see http://www.pdn.cam.ac.uk/groups/lewlab for the software; Mauritz, Esposito, Ginsburg, Kaminski, Tiffert and Lew, unpublished work).

## Supporting Information

Text S1 Description of FRET induced by molecular crowding and of the data analysis
(0.15 MB PDF)
