# Supplementary material for: FRET Imaging of Hemoglobin Concentration in Plasmodium falciparum-Infected Red Cells
Source: PLoS One. 2008 Nov 21;3(11):e3780. doi: 10.1371/journal.pone.0003780 (PMC2582953; doi:10.1371/journal.pone.0003780)
Supplement: Text S1 — Description of FRET induced by molecular crowding and of the data analysis (0.15 MB PDF) [file pone.0003780.s001.pdf]

Text S1

FRET and molecular crowding

Förster resonance energy transfer is the non-radiative transfer of energy from a donor fluorophore to an acceptor chromophore mediated by a long-range dipole-dipole interaction [1,2]. Figure 2 A shows that the fraction of quanta transferred from a donor to an acceptor – the FRET efficiency (E) – drastically depends on the inter-chromophore distance (R):

$$E = \left[1 + \left(R/R_0\right)^6\right]^{-1} \qquad \text{(S1)}$$

R_0 – the Förster distance - is the distance at which 50 % of quanta are transferred. Typical fluorophores used as FRET pairs (*e.g.*, cyan- and yellow- fluorescent proteins or green fluorescent protein and Cy3) exhibit Förster distances around 5 nm. Because of the strong dependence on intra-molecular distances within the 10 nm range, i.e., the range of protein dimensions, FRET is often used in biology to quantify protein-protein interactions [3].

Fluorescence emission is a stochastic process that follows an exponential decay distribution (see Fig. 1 B), characterized by a mean time constant usually described as the fluorescence lifetime (τ) [1,4]. The fluorescence lifetime is a molecular characteristic of a fluorophore that is considered comparatively insensitive to concentration over the physiological concentration ranges of typical biomolecules. The fluorescence lifetime is also independent from the geometrical characteristics of the detection system. FLIM is thus a robust technique for the quantitative mapping of biophysical properties of the fluorophore environment through changes in fluorescence lifetime. For instance, FRET shortens the fluorescence lifetime of a

donor fluorophore (see Fig. 1 *B*, *dashed lines*) and, indeed, FRET imaged by FLIM is one of the most robust techniques to detect protein-protein interaction with comparatively high spatial resolution [5].

At high concentrations (millimolar range) of acceptor fluorophore, a freely diffusing acceptor has a significant probability to be in close proximity to a donor fluorophore (within twice the Förster distance). Therefore, energy transfer can occur also in the absence of specific direct interaction of the two molecules; in this case, the fluorescence decay of the donor follows a stretched exponential decay [1,2]:

$$I(t) = e^{-\frac{t}{\tau_0} - 2\frac{A}{A_0}\left(\frac{t}{\tau_0}\right)^{1/2}} \qquad \text{(S2)}$$

where A is the concentration of the acceptor and A_0 is the critical concentration, defined as the concentration at which 76 % of energy is transferred. Eq. S2 is valid if there is no resonance energy transfer between donor molecules, as expected at low donor concentrations. It is important to stress that there are two different definitions of critical concentration conventionally referred to as A_0 and C_0 [1].

The critical concentration A_0 is related to the Förster distance by the following relation, where N is the Avogadro number:

$$A_0 = 1500\pi^{-3/2} N^{-1} R_0^{-3} \approx 447 R_0^{-3} \qquad \text{(S3)}$$

For A_0 to be derived in millimolar units R_0 has to be expressed in nanometres. The Förster distance depends on the donor-acceptor spectral overlap integral (J) that is defined as [1]:

$$J(\lambda) = \left(\int_0^{\infty} F_D(\lambda)\varepsilon_A(\lambda)\lambda^4 d\lambda\right) \Big/ \left(\int_0^{\infty} F_D(\lambda) d\lambda\right) \qquad \text{(S4)}$$

where F_D is the emission spectrum of the donor and ε_A is the molar extinction coefficient of the acceptor. When the wavelength is expressed in nanometres, the Förster distance is obtained in nanometres from the following equation:

$$R_0 = 0.0211\left(\kappa^2 Q J n^{-4}\right)^{1/6} \qquad \text{(S5)}$$

κ^2 is an orientation factor equal to 2/3 assuming rotational freedom of the molecules, Q is the quantum yield of the donor fluorophore and n (~1.4) is the refractive index of a red blood cell cytoplasm.

Because of the high molar extinction coefficient of Hb that extend to the visible region [6], the spectral overlap between any donor fluorophore emitting below 600 nm and Hb is not negligible (Fig. 1 C). Calcein was the donor chosen for the present experiments because its emission peak is distant to the Hb absorption peaks. It provides a quantum yield of ~0.38 [7] in sodium phosphate buffer (pH = 7.4) and its fluorescence lifetime is about 4 ns. For lower quantum yields – likely to happen in cell cytosol - R_0 and A_0 will be lower and higher, respectively, of the values computed by numerical estimation.

The Förster distance for the haeme-calcein pair was estimated to be about 4.1 nm by numerical integration of eq. S4 and substituting in eq. S5. The critical concentration of the haemoglobin tetramer ($[Hb]_0$) can thus be estimated to be about 1.7 mM.

Data analysis

Assuming the variations of quantum yield of the fluorophore proportional to the variations in the average fluorescence lifetime, the average fluorescence lifetime of calcein in the presence of haemoglobin can be predicted by the following equation [1,8]:

$$\langle \tau([Hb]) \rangle \approx \tau_0 \left\{ 1 - \sqrt{\pi} \frac{[Hb]}{[Hb]_0} e^{\left(\frac{[Hb]}{[Hb]_0}\right)^2} \left[1 - erf\left(\frac{[Hb]}{[Hb]_0} \right) \right] \right\} \quad \text{(S6)}$$

Therefore, fluorescence lifetime imaging of calcein can provide a direct estimation of [Hb]. The average lifetime was obtained by fitting scatter, IRF, background and a three exponential function with SPCImage in order to reliably fit the experimental data. At the typical photon counts obtained (~300 count per pixel), high spatial binning (5x5) was required. Furthermore, when Eq. S6 was fitted to the calibration data shown in figure 3A-B, an apparent critical concentration of 2.9 ± 0.1 mM was estimated. This was assumed to depend on the very high quenching of calcein in the RBC, that cause the measured fluorescence lifetime in physiological condition (~ 250 ps) to be in the same order of magnitude of the instrument response function (IRF, ~ 170 ps). Therefore, eq. S2 was fitted by iterative reconvolution to regions segmented by the phasor approach [9] including in the data fitting the presence of scatter (arbitrarily set as a 10 ps decay), background, unquenched calcein variable offset and IRF broadness.

A synthetic IRF was generated by the following equation:

$$IRF(t,w) = k\begin{bmatrix} \frac{1}{w\sqrt{\pi}}\exp\left[-2\left(\frac{t-t_0}{w}\right)^2\right]+ \\ 0.09\exp\left(\frac{t_0-t-0.4ns}{1.35ns}\right)+ \\ 0.05\exp\left(\frac{t_0-t-1.05ns}{1.35ns}\right) \end{bmatrix} \qquad \text{(S7)}$$

were all parameters were determined fitting an experimental IRF that was generated imaging light scattered by a microscopy glass-slide; the broadness of the Gaussian component (w) was fitted to the TCSPC data and k is a normalization factor.

Iterative reconvolution provided a more realistic critical concentration value of 1.70 ± 0.02 mM and an optimal balance between spatial resolution and robust quantification.

We note that a fluorophore with a larger Förster distance with the haeme chromophore compared with calcein could be beneficial in order to shift the critical concentration at higher values. However, it was not possible to find a fluorophore that could combine the advantages of an acetoxymethyl derivative (for non-invasive staining of living cells) providing the adequate spectral properties and availability of the non AM variant for in vitro calibration.

Molecular fractions

Let consider the fluorescence decay of calcein in the presence of molecular crowding and in the presence of an amount of non-quenched calcein molecules (c ≠ 0):

$$I(t) = \exp\left(-t/\tau\right)\left[c + a\exp\left(-2\,[Hb]/[Hb]_0\,\sqrt{t/\tau}\right)\right] \qquad \text{(S8)}$$

The equation above is eq. 1 when scatter and IRF are neglected. The weight of the fluorescence decay for the non-quenched calcein is equal to:

$$w_{CA} = c\tau \qquad \text{(S9)}$$

The weight of the fluorescence decay for the quenched calcein is equalt o:

$$w_{CA-Hb} = a\tau\left\{1 - \sqrt{\pi}\,{}^{[Hb]}\!/_{[Hb]_0}\, Exp\left({}^{[Hb]^2}\!/_{[Hb]_0^2}\right) erfc\left({}^{[Hb]}\!/_{[Hb]_0}\right)\right\} \qquad \text{(S10)}$$

Therefore eq. 2 can be inferred by computing $f_{Ca\text{-}Hb} = w_{CA\text{-}Hb} / (w_{CA\text{-}Hb} + w_{CA\text{-}Hb})$.

Reference list

1. Lakowicz JR (1999) Principles of Fluorescence Spectroscopy. New York: Kluwer Academic/Plenum Publishers.
2. Förster T (1948) Zwischenmolekulare Energiewanderung und Fluoreszenz. 437: 55-75.
3. Wouters FS, Verveer PJ, Bastiaens PI (2001) Imaging biochemistry inside cells. Trends in Cell Biology 11: 203-211.
4. Esposito A, Wouters FS, Bonifacino JS, Dasso M, Harford JB, et al. (2004) Fluorescence Lifetime Imaging Microscopy. Current Protocols in Cell Biology.
5. Pelet S, Previte MJ, So PT (2006) Comparing the quantification of Forster resonance energy transfer measurement accuracies based on intensity, spectral, and lifetime imaging. JBiomedOpt 11: 34017.
6. Horecker BL (1943) The absorption spectra of hemoglobin and its derivatives in the visible and near infra-red regions. J Biol Chem 148: 173-183.
7. Beghetto C, Renken C, Eriksson O, Jori G, Bernardi P, et al. (2000) Implications of the generation of reactive oxygen species by photoactivated calcein for mitochondrial studies. Eur J Biochem 267: 5585-5592.
8. Bennett RG (1964) Radiationless intermolecular energy transfer. I. Singlet -> Singlet tranfer. The Journal of Chemical Physics 41: 3037-3040.
9. Digman M, Caiolfa VR, Zamai M, Gratton E (2007) The Phasor approach to fluorescence lifetime imaging analysis. Biophys J.
